# Effect of Collar Diameter and Simulated Aging on the Orthogonal Load Resistance of Orthodontic Miniscrews

**DOI:** 10.3390/ma19020262

**Published:** 2026-01-08

**Authors:** Maria Francesca Sfondrini, Giuseppe Merlati, Maurizio Pascadopoli, Letizia Valceschini, Simone Ricchio, Mattia Maria Torchia, Leonardo Del Corso, Andrea Scribante

**Affiliations:** 1 Unit of Orthodontics and Pediatric Dentistry, Section of Dentistry, Department of Clinical, Surgical, Diagnostic and Pediatric Sciences, University of Pavia, 27100 Pavia, Italy; simonericchio79@gmail.com (S.R.); mattiamaria.torchia01@universitadipavia.it (M.M.T.); leonardo.delcorso01@universitadipavia.it (L.D.C.); andrea.scribante@unipv.it (A.S.); 2 Dental Materials Unit, Section of Dentistry, Department of Clinical, Surgical, Diagnostic and Pediatric Sciences, University of Pavia, 27100 Pavia, Italy; giuseppe.merlati@unipv.it (G.M.); letizia.valceschini@unipv.it (L.V.)

**Keywords:** miniscrew, orthodontics, diameter, fracture, thermocycling, autoclaving

## Abstract

The use of miniscrews as Temporary Skeletal Anchorage Devices (TSAD) in orthodontics has allowed clinicians to perform challenging tooth movements by dissipating undesired forces into the bone structure; thus, avoiding unwanted movement of the adjacent teeth. It is essential for miniscrews to be highly resistant to fracture during clinical use. While many studies have analysed torsional loads, none have measured the changes in flexural and bending strength of miniscrews before and after an ageing process. This study aims to analyse the resistance to orthogonal forces of miniscrews with different diameters, focusing on both new and aged materials, the latter subjected to thermocycling and autoclaving laboratory processes to simulate a 3- and a 6-month exposure to the oral environment. A total of 105 pristine miniscrews have been tested; specimens were divided into seven groups based on the different endosseous body diameters. Each group was further subdivided into three subgroups, according to the simulated ageing of the miniscrews (intact, 3 months of ageing and 6 months of ageing, respectively). An Instron Universal Testing Machine has been used to measure deflection at 0.1 mm and 0.2 mm, as well as maximum load at fracture. The results evidenced that miniscrews respond differently to cutting forces; in particular, the resistance to orthogonal loads increases as the diameter of the miniscrews increases. Linear regression analysis revealed a significant influence between all the dependent variables—maximum load, 0.1 mm deflection load, and 0.2 mm deflection load—and the independent variables, such as diameter and thermocycling (*p* < 0.05). Both new and aged miniscrews are suitable for orthodontic and orthopaedic loads; moreover, ageing up to 6 months does not seem to significantly decrease the resistance to shear forces for the same diameter. Linear regression analysis of the miniscrews subjected to experimental ageing showed a slight but significant decrease in resistance to orthogonal loading.

## 1. Introduction

Interceptive orthodontic treatment in early childhood capitalises on a critical developmental window to create adequate space for the eruption of permanent teeth. Early intervention may significantly reduce the need for future orthodontic procedures, dental extractions, or subsequent orthognathic surgery [1,2,3,4,5,6,7,8,9].

Transverse maxillary deficiency, for instance, is a prevalent orthodontic condition that can contribute to asymmetrical facial growth, mandibular retrusion, temporomandibular joint disorders, and a predisposition to oral breathing [10]. The optimal timing for the treatment of maxillary transverse deficiency, as well as the most appropriate method of maxillary expansion, remains a subject of ongoing debate. Various approaches have been employed to assess the relationship between palatal suture expansion and several diagnostic indicators [11]. Among the most significant of these are chronological age [12] and skeletal age [13].

As patients pass from adolescence to adulthood, greater force is required to initiate the process of opening the midpalatal suture [14]. The pursuit of a non-surgical option for maxillary transverse deficiency in adolescent and adult patients led to the development of the Miniscrew-Assisted Rapid Palatal Expansion (MARPE) technique [15,16]. This methodology aims to achieve maximal expansion while concurrently minimising deleterious effects on the posterior teeth [17,18]. Furthermore, it significantly increases the volume of the upper airways in adults [19]. Notwithstanding the evident benefits of this procedure, judicious patient selection is imperative, requiring a preoperative CT to evaluate the morphology of the suture and bone thickness, with the objective of averting the occurrence of maxillary fractures arising from stress concentration during the active expansion phase [20].

Temporary Skeletal Anchorage Devices (TSADs) have been increasingly employed in the orthodontic practice mainly to manage undesired orthodontic forces and to provide skeletal anchorage [21,22]. If compared with traditional devices, miniscrews provide significantly better outcomes in terms of stability, alignment, gingival health, masticatory function, treatment effectiveness, and patient satisfaction, with fewer adverse reactions and a success rate from 75% to 90% [23,24].

Miniscrews currently represent the most widespread form of TSADs; these biocompatible devices are employed as bone fixation screws for mechanical retention in both orthodontic and orthopaedic treatments [6,25]. Miniscrews are particularly useful in those cases where the patients are uncooperative or when the conventional anchorage methods are insufficient to properly correct the malocclusion [26,27,28]. Numerous in vitro studies have been conducted to analyse various mechanical parameters influencing fracture risk. Specifically, factors such as removal torque [29] and plastic deformation [30] have been systematically evaluated to better understand their impact on miniscrew performance and reliability.

However, there is limited scientific evidence in that field; it appears that orthodontic miniscrews failure rates may be related to the insertion sites, with an overall high rate of failure (1.3–16.4%). The major risk factor for failure is root contact [31,32]. The most significant complications are loss of stability [33] and fracture [34], the latter typically being caused by excessive torsional forces exerted during insertion or removal procedures [35]. Whereas insertion/removal torque is the primary cause of miniscrew breakages [36], it should be acknowledged that a significant percentage of fractures is also due to the orthogonal loads [37,38]. The combined application of miniscrews in orthodontic appliances such as the rapid palatal expander (RPE) [39] or the Herbst appliance [40] has made the analysis of the orthogonal loads of increasing interest.

The objective of the present study is two-fold: firstly, to assess the feasibility of reusing orthodontic miniscrews within the same patient after a first clinical use, and secondly, to determine whether mechanical changes occur that may increase the risk of fracture.

In the present study, miniscrews of different diameters were tested, including both intact and experimentally aged ones. The latter were subjected to laboratory procedures such as thermocycling and autoclaving in order to simulate 3- and 6-month exposure inside the oral cavity.

## 2. Materials and Methods

The study investigated 105 pristine miniscrews divided into 7 groups of 15 samples, according to the endosseous body diameter (Table 1).

Each group was further divided into three subgroups of five samples each, based on the ageing procedure used. The first subgroup was subjected to a 3-month ageing treatment, while the second subgroup was subjected to a 6-month ageing treatment; on the contrary, the third subgroup was not treated and served as a control. To simulate the oral environment, a thermocycling laboratory technique was employed (Thermocycler FCA14 (ICR snc, Cava Manara, Italy)); the protocol of the test was an alternated and repeated immersion of the samples in 5 °C and 55 °C for 30 s, with a transfer time of 10 s (ISO/TS 11405:2015) [41].

Samples were exposed to each tank for 30 s to replicate the sudden temperature changes that occur in the oral environment [42]. The study involved two simulations of 3- and 6-month exposure to the oral environment. Since it has been estimated that 1 year of exposure inside the oral cavity is equivalent to about 10,000 cycles (30 thermic excursions per day) [43], the 3-month simulation consisted of 2500 cycles, while the 6-month simulation consisted of 5000 cycles. Before being tested, the thermocycled samples were subjected to a standard autoclaving cycle, and the procedure was repeated after the first 2500 cycles. The miniscrews tested at 5000 cycles were autoclaved twice. The sterilisation method employed in this study involved the exposition of the materials to a steam-saturated environment at 121 °C for 15–20 min [44,45]. Table 2 presents the laboratory procedures followed for the realisation of the present study.

**Figure 1:**
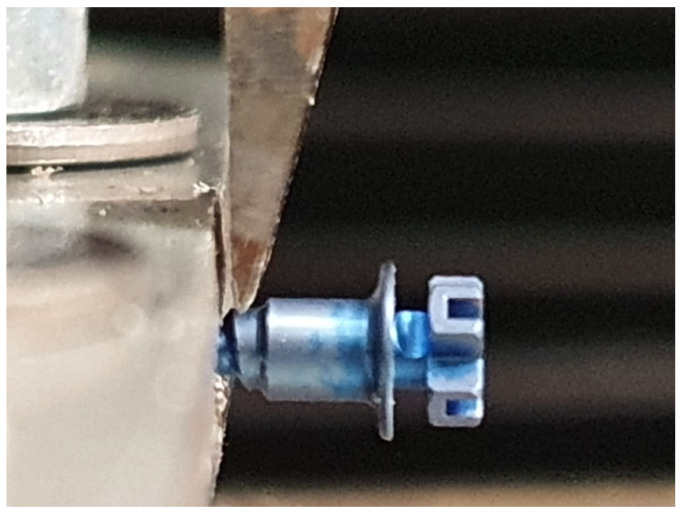
Point of application of the force. Span length: 0.5 mm from the tip to the block.

An Instron Universal Testing Machine (Model 3343, Instron Corporation, Canton, MA, USA) was used to apply the orthogonal load at constant orientation. The machine has been set by using Bluehill 2 software (Instron Industrial Products, Grove City, PA, USA) to record the load forces required to cause a deflection of 0.1 mm and 0.2 mm, other than the point of breakage (maximum load). The load progression rate was set at 1 mm/minute [37]. The 0.1 mm and 0.2 mm deflection thresholds were chosen to reflect clinically meaningful limits of elastic deformation in miniscrews, distinguishing minor reversible displacement from micromobility levels that may jeopardise anchorage stability. To ensure the proper immobilisation of the miniscrews during the experiment, two steel plates were arranged and connected to each other and then engraved, thus obtaining a mechanical engagement between the plates and the specimen body. The miniscrews were inserted into the locking system, which was then closed without torque application. This was performed to prevent torsional forces from affecting the shear test results [37]. The load application point is identified at the passage between the body and the collar of the miniscrew, with a span length of 0.5mm from the tip to the block (Figure 1). Furthermore, shear load was applied at a constant orientation.

The collected data were analysed using R software (version 3.1.3, R Development Core Team, R Foundation for Statistical Computing, Wien, Austria). Descriptive statistics, including mean, standard deviation, minimum, median, and maximum, have been calculated for the 21 groups. The normality of the data was assessed by using the Kolmogorov–Smirnov test. ANOVA followed by Tukey’s post hoc test was performed to identify eventual differences among the force values of the different groups. Linear regression analysis was performed to assess the relationship between all the dependent variables (maximum load, 0.1 mm deflection load and 0.2 mm deflection load) and the independent variables (endosseous body diameter and thermocycling) considered in the current study. All statistical tests were considered significant at *p* < 0.05.

## 3. Results

**Figure 2:**
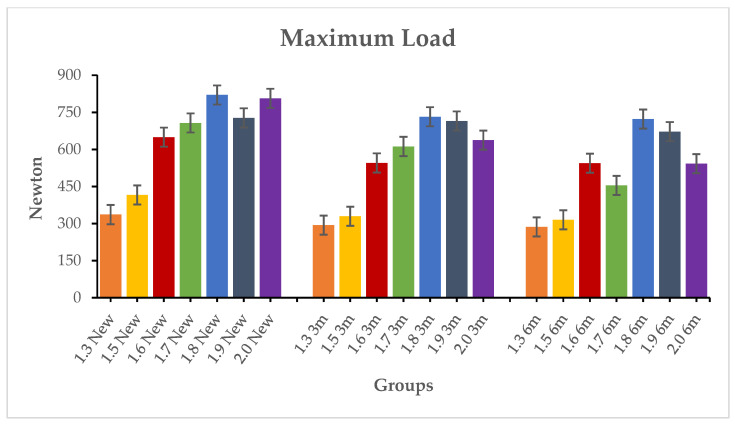
Comparison between the maximum load of the samples in the new types, 3 months aged and 6 months aged.

**Figure 3:**
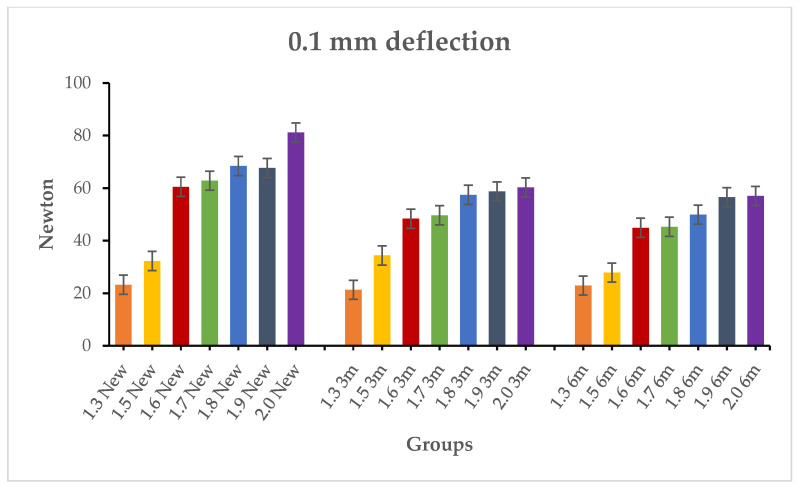
Comparison between the 0.1 mm deflection load of the samples in the new types, 3 months aged, and 6 months aged.

**Figure 4:**
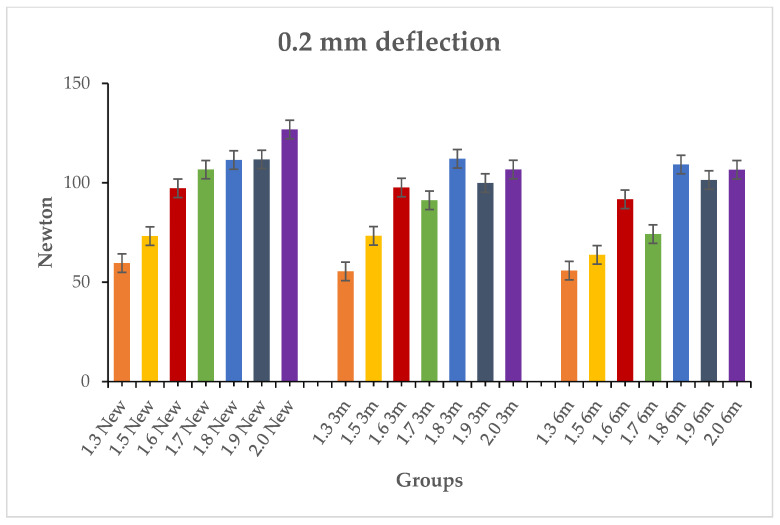
Comparison between the 0.2 mm deflection load of the samples in the new types, 3 months aged and 6 months aged.

The results of inferential statistics revealed significant differences within the three groups for the maximum load, 0.1 mm deflection and 0.2 mm deflection, considering that, in general, higher loads corresponded to higher diameters (*p* < 0.05). Intergroup analysis was conducted comparing the miniscrews with the same diameter in the three groups. The results of Tukey’s test showed no significant differences for the three variables (*p* > 0.05). Table 3, Table 4 and Table 5 present the descriptive and inferential statistics of the force values recorded in the seven subgroups. The tables include mean, standard deviation, minimum, median, and maximum values for the three variables of the study: maximum loading (Table 3), 0.1 mm deflection load (Table 4), and 0.2 mm deflection load (Table 5). Figure 2, Figure 3 and Figure 4 present the graphical representation of the descriptive statistics for the three variables of the study.

Linear regression analysis (Table 6) reveals a significant influence between all the dependent variables—maximum load, 0.1 mm deflection load, and 0.2 mm deflection load—and the independent variables such as diameter and thermocycling (*p* < 0.05).

**Figure 5:**
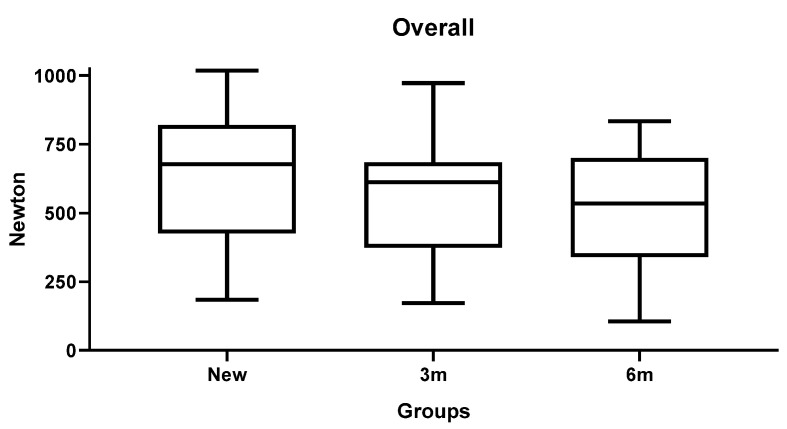
Cumulative boxplot showing a slight reduction in maximum forces after simulated ageing.

The cumulative box plots illustrate the reduction in maximum forces consequent to the simulated ageing process (Figure 5).

## 4. Discussion

The use of miniscrews has become increasingly common in orthodontics, particularly in procedures that involve heavy loads and produce significant orthogonal forces, such as rapid palatal expansion [39]. However, a significant percentage of fractures is linked to these orthogonal loads [37]. Therefore, it is important to evaluate the reusability of miniscrews in such cases. Despite manufacturers selling miniscrews for single use, several authors have studied the possibility of reusing them, analysing the mechanical capacity of aged mini screws with a particular focus on their resistance to fracture torque (FT) as a function of diameter [46,47,48]. Indeed, the reuse of medical devices in the same patient after sterilisation is a common practice in various medical fields [49]. In orthodontics, some authors suggested the possible reuse of wires [50,51], bands, and brackets [52]. Reusing mechanically and functionally efficient materials permits to both reduce the treatment costs and to prevent the waste of high-quality resources [47]. In the context of miniscrews, it has been demonstrated that their reinsertion in the same patient does not result in a significant loss of primary stability or changes in mechanical properties [53,54]. Furthermore, it has been established that autoclave sterilisation does not cause any change in their fracture torque [55]. Conversely, the application of dry heat sterilisation appears to exert negative influences on the mechanical properties of miniscrews, particularly with regard to their fracture torque [56].

The extant literature on the subject includes two studies concerning the evaluation of the orthogonal load in relation to the diameter [37] and material [38] of the miniscrews analysed. However, none of these studies consider the submission of miniscrews to ageing. The paucity of research on the variation in the mechanical capacity of aged miniscrews in relation to orthogonal fracture loads and diameter is the justification for the objective of this study.

That is, to analyse the resistance to orthogonal forces of miniscrews with different diameters, focusing on both new and aged materials, the latter subjected to thermocycling and autoclaving laboratory processes to simulate a 3- and a 6-month exposure to the oral environmental conditions. The analysis performed in the present study shows that the force required to cause a fracture increases as the device diameter increases. Intragroup significance analysis of load values at 0.1 mm and 0.2 mm deflection (Table 4 and Table 5) also reveals that a major force is required to achieve the same deflection as the device diameter increases. The linear regression analysis revealed a statistically significant relationship between the diameter of the device and the miniscrews resistance under maximum load, 0.1 mm deflection load and 0.2 mm deflection load. Therefore, it can be concluded that an increase in the diameter of the device is associated with an increase in the resistance of the miniscrews under maximum load, 0.1 mm deflection load, and 0.2 mm deflection load. These observations confirm the association between diameter, fracture resistance and deflection under orthogonal loads, as previously demonstrated [37]. During clinical use, miniscrews are primarily subjected to orthopaedic loads, which typically do not exceed 600 g [25], equivalent to 5.88 N. Based on the results of the study, it was found that a minimum load of 10.00 N is required to significantly alter the device. This data is recorded during a load test that caused a deflection of 0.1 mm on a single new miniscrew with a diameter of 1.3 mm, which is the smallest sample examined (Table 4). These results confirm that even the smallest miniscrews currently available (1.3 mm) can support loads far above physiological ones without compromising mechanical resistance, based on a 6-month ageing procedure. Those findings are in accordance with the results obtained from research about the failure rate of miniscrews [14,57] and support recent evidence suggesting that sterilised miniscrews may maintain sufficient mechanical integrity for reuse within the same patient; however, given the increased failure rates reported in clinical studies of reused devices, such practice should be approached with caution and validated through further clinical trials [58,59]. To completely understand the aim of the study, and considering its potential employment as a guide for future research in this field, it is important to evidence the limitations of the proposed study. In particular, the ageing of the specimens and the tests for shear force resistance have been performed in a laboratory, thus not in a clinical setting. For this reason, autoclaved miniscrews have not been exposed to prior loads, insertion torque, microcracks or bone interaction forces, in contrast to the conditions that occur in the clinical environment. Furthermore, the ageing process adopted in this study does not simulate in any way the mechanical fatigue (chewing, bone remodelling, micro-movements) to which miniscrews are subjected inside the oral cavity. In addition, it is important to consider that the study only examined seven types of miniscrews, which is a relatively small sample when compared to the wider range available on the market. The lack of uniformity among products from different manufacturers may have resulted in an increase in the standard deviation of certain groups, thus influencing the heterogeneity of the results. Furthermore, length, thread design, collar architecture and surface texture of miniscrews differ between the various brands considered in this study, and could contribute to variations in resistance to orthogonal forces. Additional in vitro and clinical experimentation is required to enhance the current understanding of miniscrew resistance to shear forces. In vitro experiments with a larger sample of devices and longer ageing times are necessary. The implementation of a microstructural analysis or microscopic surface inspection would facilitate the determination of the fatigue strength of the miniscrews. Additionally, clinical studies on patients are needed to overcome the limitations of in vitro experimentation, to evaluate clinical failures, and to accurately analyse the causes of device breakage. From a clinical perspective, it would be appropriate to assess the failure rate of autoclaved miniscrews, correlating it with the type of orthodontic device used, the patient’s hygiene habits and the forces transmitted.

## 5. Conclusions

The results showed that the mechanical resistance of the specimens to orthogonal loads increases linearly with their diameter. Therefore, the diameter significantly affects the clinical performance of the miniscrews. All the tested miniscrews have been found to be suitable for orthodontic and orthopaedic loads. Laboratory-induced 3- and 6-month ageing did not lead to a significant reduction in the resistance to shear forces for equal diameters. Based on these observations, it can be hypothesised that in the event of loss of an orthodontic miniscrew and clinical failure, the device could be autoclaved and reused on the same patient up to two times and up to 6 months, even for orthopaedic forces, regardless of its diameter.

## Figures and Tables

**Table 1:** Miniscrews were adopted in the study.

Group	Name	Manufacturer	Location	Diameter	Length	Composition
1	Medicon Aarheus Screw	Medicon EG	Tuttlingen, Germany	1.3 mm	12.3 mm	Titanium Grade 5 (Ti-6Al-4V)
2	HDC Spider Screw	HDC Srl	Thiene, Italy	1.5 mm	10.0 mm	Titanium Grade 5 (Ti-6Al-4V)
3	Medicon Aarheus Screw	Medicon EG	Tuttlingen, Germany	1.6 mm	10.2 mm	Titanium Grade 5 (Ti-6Al-4V)
4	Ortho Easy Forestadent	Forestadent	Pioltello, Italy	1.7 mm	10.0 mm	Titanium Grade 5 (Ti-6Al-4V)
5	3M OrthoImplant	3M Italia Spa	Pforzheim, Germany	1.8 mm	10.0 mm	Titanium Grade 5 (Ti-6Al-4V)
6	HDC Spider Screw	HDC Srl	Thiene, Italy	1.9 mm	10.0 mm	Titanium Grade 5 (Ti-6Al-4V)
7	Kristal Storm	Kristal Srl	Trezzano sul Naviglio, Italy	2.0 mm	10.0 mm	Titanium Grade 5 (Ti-6Al-4V)

**Table 2:** Chronological sequence of procedures adopted on each of the three study subgroups.

Group	Miniscrew Name and Diameter	Subgroup	Thermocycling ^1^	Autoclaving ^2^	Thermocycling ^1^	Autoclaving ^2^	Instron Test ^3^
		Control					X
1	Medicon Aarheus Screw (1.3 mm)	3 months ageing	X	X			X
		6 months ageing	X	X	X	X	X
		Control					X
2	HDC Spider Screw (1.5 mm)	3 months ageing	X	X			X
		6 months ageing	X	X	X	X	X
		Control					X
3	Medicon Aarheus Screw (1.6 mm)	3 months ageing	X	X			X
		6 months ageing	X	X	X	X	X
		Control					X
4	Ortho Easy Forestadent (1.7 mm)	3 months ageing	X	X			X
		6 months ageing	X	X	X	X	X
		Control					X
5	3M OrthoImplant (1.8 mm)	3 months ageing	X	X			X
		6 months ageing	X	X	X	X	X
		Control					X
6	HDC Spider Screw (1.9 mm)	3 months ageing	X	X			X
		6 months ageing	X	X	X	X	X
		Control					X
7	Kristal Storm (2.0 mm)	3 months ageing	X	X			X
		6 months ageing	X	X	X	X	X

^1^ 2500 cycles, tanks at 55 °C and 5 °C, permanence in the tanks for 30 s with a transfer time of 10 s. ^2^ 15–20 min exposure to a steam saturated environment at 121 °C. ^3^ Shear strength test set in compression with blade speed equal to 1 mm/min. X: treatment performed.

**Table 3:** Descriptive statistics of maximum load in the different groups.

Diameter	Type	Mean	SD	Min	Mdn	Max	Intragroup Significance ^1^	Intergroup Significance ^1^
1.3		336.36	123.18	184.56	318.97	525.62	A	A
1.5		415.56	22.93	393.61	406.09	452.47	A, B	A
1.6		649.35	48.80	570.41	650.89	699.23	B, C, D	A
1.7	new	706.86	184.84	425.39	822.42	849.79	B, C, D	A
1.8		820.29	78.66	695.57	868.92	876.08	D	A
1.9		727.42	168.72	584.07	684.95	1018.45	D	A
2.0		806.81	25.95	765.23	815.18	832.91	D	A
1.3		293.65	77.19	186.12	314.71	373.22	A	A
1.5		329.61	156.53	171.62	268.51	572.70	A, B	A
1.6		545.46	203.40	373.20	456.99	840.02	A, B, C	A
1.7	3 months	611.96	64.66	508.26	619.60	685.62	B, C	A
1.8		731.98	137.62	624.47	684.74	973.01	C	A
1.9		715.19	180.13	466.15	721.19	934.45	C	A
2.0		637.26	46.74	588.95	633.63	703.71	C	A
1.3		286.83	147.15	105.57	295.17	442.49	A	A
1.5		315.50	156.21	183.54	292.90	572.33	A, B	A
1.6		544.13	126.21	340.06	580.94	682.72	A, B, C	A
1.7	6 months	454.61	219.68	289.50	360.08	833.64	A, B, C	A
1.8		722.68	21.29	700.67	725.08	745.77	C	A
1.9		671.98	122.46	500.43	704.24	791.27	C	A
2.0		542.24	15.46	524.79	539.80	559.10	A	A

^1^ Letter-based significance visualisation: means presenting at least a common capital letter, which does not show significant differences (*p* > 0.05). Significance is divided into intragroup and intergroup comparisons. Intergroup comparisons are between miniscrews of the same diameters.

**Table 4:** Descriptive statistics of the 0.1 mm deflection load in the different groups.

Diameter	Type	Mean	SD	Min	Mdn	Max	Intragroup Significance ^1^	Intergroup Significance ^1^
1.3		23.23	16.09	10.00	18.40	50.92	A	A
1.5		32.26	4.38	28.76	30.79	39.77	A, B	A
1.6		60.50	5.30	51.85	61.72	64.89	B, C	A
1.7	new	62.82	24.80	24.65	76.23	84.07	B, C	A
1.8		68.41	6.48	57.39	70.08	74.63	C	A
1.9		67.64	21.82	51.04	61.00	105.45	C	A
2.0		81.15	1.88	78.39	81.72	83.17	C	A
1.3		21.28	9.40	10.39	18.73	32.04	A	A
1.5		34.38	17.15	17.36	27.65	58.85	A, B	A
1.6		48.37	6.60	37.60	50.04	55.53	A, B	A
1.7	3 months	49.66	13.08	38.60	41.94	67.47	A, B	A
1.8		57.44	17.29	28.79	59.65	71.15	B	A
1.9		58.74	17.27	34.37	61.70	79.70	B	A
2.0		60.28	5.74	54.57	59.16	69.50	B	A
1.3		22.90	6.97	14.67	25.11	29.81	A	A
1.5		27.85	6.15	21.32	28.51	35.81	A, B	A
1.6		44.91	19.30	16.26	49.37	62.66	A, B	A
1.7	6 months	45.28	14.95	27.28	47.96	61.46	A, B	A
1.8		49.91	12.77	32.16	47.65	64.50	A, B	A
1.9		56.54	7.73	48.98	56.54	68.71	B	A
2.0		57.00	8.90	46.10	54.74	68.28	B	A

^1^ Letter-based significance visualisation: means presenting at least a common capital letter, which does not show significant differences (*p* > 0.05). Significance is divided into intragroup and intergroup comparisons. Intergroup comparisons are between miniscrews of the same diameters.

**Table 5:** Descriptive statistics of the 0.2 mm deflection load in the different groups.

Diameter	Type	Mean	SD	Min	Mdn	Max	Intragroup Significance ^1^	Intergroup Significance ^1^
1.3		59.53	23.17	31.38	57.03	95.63	A	A
1.5		73.17	5.39	68.68	71.42	82.43	A, B	A
1.6		97.22	5.59	88.12	98.64	101.74	B, C	A
1.7	new	106.61	28.66	62.45	121.73	131.50	B, C	A
1.8		111.41	7.09	99.49	112.99	118.37	C	A
1.9		111.74	25.07	93.02	103.92	155.19	C	A
2.0		126.79	2.02	124.09	127.68	128.88	C	A
1.3		55.39	13.25	40.11	56.52	74.32	A	A
1.5		73.32	28.32	38.98	65.91	112.98	A, B	A
1.6		97.56	6.26	90.29	96.93	106.50	B, C	A
1.7	3 months	91.18	11.11	80.27	92.68	107.97	B, C	A
1.8		112.10	16.31	96.19	107.24	131.66	C	A
1.9		99.86	12.65	80.38	99.03	112.26	C	A
2.0		106.62	7.66	97.59	105.42	117.85	C	A
1.3		55.82	12.11	45.52	49.35	73.88	A	A
1.5		63.77	10.01	52.91	64.84	75.15	A, B	A
1.6		91.65	23.42	53.30	99.60	111.67	A, B, C	A
1.7	6 months	74.15	12.01	59.08	79.97	84.37	A, B, C	A
1.8		109.18	13.20	92.51	109.07	122.32	C	A
1.9		101.38	8.91	88.93	100.10	110.66	C	A
2.0		106.52	11.09	97.80	99.68	121.29	C	A

^1^ Letter-based significance visualisation: means presenting at least a common capital letter, which does not show significant differences (*p* > 0.05). Significance is divided into intragroup and intergroup comparisons. Intergroup comparisons are between miniscrews of the same diameters.

**Table 6:** Linear regression of the considered variables. *: *p* < 0.05.

Dependent Variable	Independent Variable	Significance
Maximum load	Diameter	*p* < 0.0001 *
	Thermocycling	*p* = 0.007 *
0.1 mm deflection load	Diameter	*p* < 0.001 *
	Thermocycling	*p* = 0.006 *
0.2 mm deflection load	Diameter	*p* < 0.001 *
	Thermocycling	*p* < 0.045 *

## Data Availability

The original contributions presented in this study are included in the article. Further inquiries can be directed to the corresponding authors.
